# THE COMBINED EFFECTS OF SMOKING, DRINKING, AND PHYSICAL ACTIVITIES ON THE COGNITIVE HEALTH OF OLDER ADULTS

**DOI:** 10.1093/geroni/igae098.0400

**Published:** 2024-12-31

**Authors:** Yan-Jhu Su, Elizabeth Dugan, Jaqueline Avila

**Affiliations:** University of Massachusetts Boston, Boston, Massachusetts, United States; University of Massachusetts Boston, Boston, Massachusetts, United States; University of Massachusetts Boston, Boston, Massachusetts, United States

## Abstract

**Background:**

Poor health behaviors such as smoking tobacco, alcohol consumption, and limited physical activity may be associated with poor cognitive health. Unhealthy behaviors often co-occur, and little research has examined the combined or cumulative effects of unhealthy behaviors on cognitive health. This examines how common health behaviors may co-occur and their associations with cognitive health among older U.S. adults.

**Methods:**

This cross-sectional study used 2018’s Health and Retirement Study (HRS) data, including 7,467 respondents aged 65 and older. Cognitive function scores were assessed with the Telephone Interview of Cognitive Status-Modified. Health behavior combinations were assessed, including smoking status, drinking amount, and frequency of physical activities. Linear regression was used to examine the association between different health behavior combinations and cognitive function.

**Results:**

The most common combinations were healthy ones: never smoking, no drinking, and high-level physical activity (15.47%; reference group). All combinations with a prevalence of less than 2% were combined as “other” combinations. Compared to the reference group, respondents who never smoked, had no drinking, and had no physical activities had lower cognitive function (b = -1.13, 95% CI: -1.56, -0.71). Other unhealthy combinations that impacted cognition were never-smoked, had no drink, and low physical activities; and former smokers had no drink and low physical activities.

**Conclusions:**

This cross-sectional study found that high levels of physical activity and some drinking are associated with better cognitive health, while smoking was associated with poorer cognitive health. Policies to promote physical activity across the lifespan may be promising avenues to pursue.

